# The genome of the truffle-parasite *Tolypocladium ophioglossoides* and the evolution of antifungal peptaibiotics

**DOI:** 10.1186/s12864-015-1777-9

**Published:** 2015-07-28

**Authors:** C. Alisha Quandt, Kathryn E. Bushley, Joseph W. Spatafora

**Affiliations:** Department of Botany and Plant Pathology, Oregon State University, Corvallis, OR USA; Department of Plant Biology, University of Minnesota, St. Paul, MN USA; Present address: Department of Ecology and Evolutionary Biology, University of Michigan, Ann Arbor, MI USA

**Keywords:** Secondary metabolism, Hypocreales, Mycoparasites, Lineage sorting

## Abstract

**Background:**

Two major mycoparasitic lineages, the family Hypocreaceae and the genus *Tolypocladium*, exist within the fungal order, Hypocreales. Peptaibiotics are a group of secondary metabolites almost exclusively described from *Trichoderma* species of Hypocreaceae. Peptaibiotics are produced by nonribosomal peptide synthetases (NRPSs) and have antibiotic and antifungal activities. *Tolypocladium* species are mainly truffle parasites, but a few species are insect pathogens.

**Results:**

The draft genome sequence of the truffle parasite *Tolypocladium ophioglossoides* was generated and numerous secondary metabolite clusters were discovered, many of which have no known putative product. However, three large peptaibiotic gene clusters were identified using phylogenetic analyses. Peptaibiotic genes are absent from the predominantly plant and insect pathogenic lineages of Hypocreales, and are therefore exclusive to the largely mycoparasitic lineages. Using NRPS adenylation domain phylogenies and reconciliation of the domain tree with the organismal phylogeny, it is demonstrated that the distribution of these domains is likely not the product of horizontal gene transfer between mycoparasitic lineages, but represents independent losses in insect pathogenic lineages. Peptaibiotic genes are less conserved between species of *Tolypocladium* and are the product of complex patterns of lineage sorting and module duplication. In contrast, these genes are more conserved within the genus *Trichoderma* and consistent with diversification through speciation.

**Conclusions:**

Peptaibiotic NRPS genes are restricted to mycoparasitic lineages of Hypocreales, based on current sampling. Phylogenomics and comparative genomics can provide insights into the evolution of secondary metabolite genes, their distribution across a broader range of taxa, and their possible function related to host specificity.

**Electronic supplementary material:**

The online version of this article (doi:10.1186/s12864-015-1777-9) contains supplementary material, which is available to authorized users.

## Background

Hypocreales is home to a wide array of ecologically diverse fungi. Some are devastating plant pathogens (*e.g*., *Fusarium* spp.), while others form numerous lineages of both insect pathogens (*e.g*., *Cordyceps*) and mycoparasites (*e.g*., *Trichoderma*) [1]. At the divergence of the four most derived families (Clavicipitaceae, Cordycipitaceae, Hypocreaceae, and Ophiocordycipitaceae) of Hypocreales, there was a major shift away from plant-based nutrition to either insect pathogenesis or fungal parasitism, *i.e*., mycoparasites [2]. Two major lineages of mycoparasites are found within the order, although other mycoparasites exist (*e.g*., several species of *Polycephalomyces*; [3, 4]). The first and larger of these two lineages is the family Hypocreaceae, most notable for mycoparasitic *Trichoderma* spp. used in biological control of plant pathogenic fungi, and *Trichoderma reesei E.G*. Simmons [5], the industrial workhorse for cellulase production [6, 7]. The second major lineage of mycoparasites, the genus *Tolypocladium*, is nested within the insect pathogenic family, Ophiocordycipitaceae [1, 8]. Most species of *Tolypocladium* parasitize the truffles of *Elaphomyces* [Eurotiales, Ascomycota], ectomycorrhizal fungi closely related to *Aspergillus* and *Penicillium* [9, 10]. *Tolypocladium ophioglossoides* (Ehrh. ex J.F. Gmel.) Quandt, Kepler & Spatafora is a commonly collected truffle parasite with a broad geographic distribution throughout many parts of the Northern Hemisphere [11, 12]. There are, however, a few *Tolypocladium* species that attack insects and rotifers, and based on current multigene phylogenies some of these are inferred to be reversals to insect pathogenesis [1, 8, 13]. One of these is a beetle pathogen, *T. inflatum*, which was the first source of the immunosuppressant drug, cyclosporin A [14]. Evidence from multigene studies has also shown a close phylogenetic relationship between *T. ophioglossoides* and *T. inflatum* [1].

Secondary metabolism is defined as the synthesis of often bioactive, small molecules that are not essential to the growth of an organism. Genes related to production of secondary metabolites are often clustered together in close proximity within a genome and coregulated [15]. A wide variety of secondary metabolites including the ergot alkaloids, fumonisins, and destruxins, is produced by species of Hypocreales [16–18]. Many of these metabolites are produced by nonribosomal peptide synthetases (NRPSs), which are often large, multi-modular proteins that produce short peptides frequently incorporating non-standard amino acids. NRPS modules are composed of three primary functional domains including adenylation (A), thiolation (T), and condensation (C) domains [19]. Due to their high level of amino acid and nucleotide conservation, the A-domains are frequently used to reconstruct the evolutionary histories of these genes [20, 21]. Polyketide synthases (PKSs) are another class of secondary metabolite producing enzymes that are common in fungi and are also modular in nature. They are related to fatty acid synthases [22], and assemble small bioactive molecules based on acetyl-CoA or malonyl-CoA subunits [23]. The other major classes of secondary metabolite-producing enzymes are terpene synthases and dimethylallyltryptophan (DMAT) synthases, both of which have been reported from hypocrealean taxa. Fungal secondary metabolites clusters often include genes required for regulation of expression of the gene cluster and decoration, epimerization, and transport of the mature secondary metabolite [24, 25].

Peptaibols, or peptaibiotics, are antibiotic secondary metabolites products produced by very large NRPS enzymes (up to 21,000 amino acids in length). Their name is a derivative of their structure as they are Peptides containing the uncommon non-proteinogenic amino acid, α-amino isobutryic acid (AIB), and a C-terminal amino ethanol [26]. The presence of AIB residues promotes helix formation, and several of these helices form multimeric units that in turn form voltage gated ion channels capable of inserting into cell membranes where they disrupt membrane potential causing leakiness [27, 28]. Peptaibols are produced by *Trichoderma* spp. and other members of Hypocreaceae (The Peptabiol Database [29]), leading to the proposition that they may play a role in mycoparasitism. There is at least one empirical study to support this in *Trichoderma* [30]. Further studies have found that peptaibols function, along with cell wall degrading enzymes, to synergistically inhibit new cell wall synthesis in fungal prey of *Tr. harzianum* [31–33]. Wiest et al. [34] identified and characterized the first peptaibol synthetase NRPS modular structure (a product of the *tex1* gene) from *Tr. virens* along with the 18 residue peptaibol product. Since that time, several other peptaibol NRPS genes have been identified in *Tr. virens* and other species [21, 35, 36].

Efrapeptins are another class of peptaibiotics described almost exclusively from *Tolypocladium* spp. that have antifungal and insecticidal properties [37–39]. They differ from orthodox peptaibols by the presence of a mitochondrial ATPase inhibiting C-terminal “blocking group” N-peptido-1-isobutyl-2[1-pyrrole-(1-2-α)-pyrimidinium,2,3,4,6,7,8-hexahydro]-ethylamine [40]. Eight of these, named efrapeptins A, and C-I, have been isolated from *T. inflatum* [38, 41].

Numerous genomes from species of the mycoparasitic genus *Trichoderma* (Hypocreaceae) have been sequenced [42, 43], and more recently the genomes of several insect pathogens in Hypocreales have been completed (*e.g*., *Cordyceps militaris*, *Beauveria bassiana*, *Metarhizium* spp., and *Ophiocordyceps sinensis*) [44–47], including *T. inflatum*, the beetle pathogenic congener of *T. ophioglossoides* [48]. The genomes of all of these species are rich in secondary metabolite genes and clusters, ranging from 23 to 51 secondary metabolite gene clusters per genome. Comparisons of gene content and expression are beginning to shed light on mechanisms underlying host specificity and the evolution of primary and secondary metabolism. In this study, the draft genome of the truffle parasite *T. ophioglossoides* was generated to compare the gene content and secondary metabolite content of this truffle parasite to those of closely related insect pathogens and more distantly related mycoparasites. The secondary metabolite potential of *T. ophioglossoides* is characterized with a focus on understanding the evolution of gene clusters encoding for peptaibiotics.

## Results and discussion

### Genome assembly and structure

**Fig. 1:**
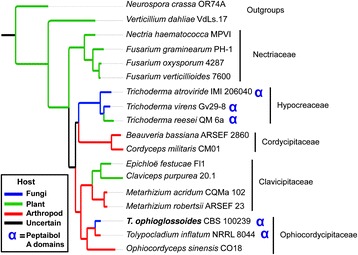
Maximum likelihood phylogeny of hypocrealean taxa analyzed in this study. RAxML tree created using concatenated alignment of 1,495 protein clusters identified in the HAL pipeline [87] with 622,497 amino acid positions and 245,180 distinct alignment patterns. Branches are colored based on host/nutritional association. Both pathogens and saprobes of plant material are colored green. Species that possess peptaibol A-domains are denoted by a blue ‘α’

**Fig. 2:**
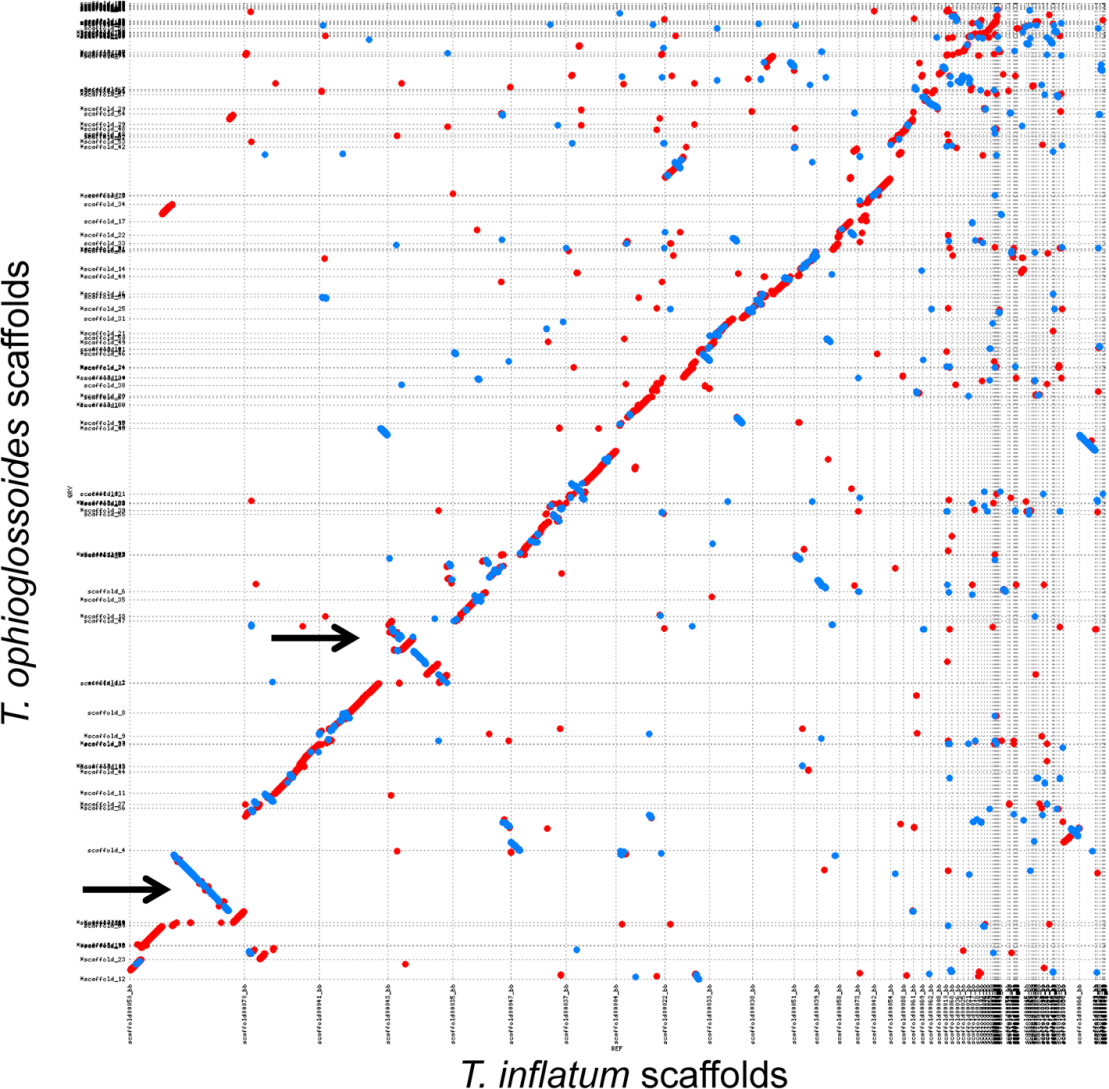
Whole genome nucleotide alignment of the *Tolypocladium ophioglossoides* and the *T. inflatum* genomes. Mummerplot visualization of nucmer alignment of all 172 unmasked *T. ophioglossoides* scaffolds against the reference 101 *T. inflatum* scaffolds. Red lines represent sequence aligning in the same direction while blue lines represent inversions. The black arrows point out the major inversions between the two genomes

The draft genome assembly of the *T. ophioglossoides* CBS 100239 is approximately 31.2 megabases (Mb) and is assembled on 172 scaffolds. The assembly, with an n50 of 668,222 base pairs (bp), captures the majority of gene space and synteny [Table 1], in spite of the exclusive use of short read technology. In addition, the Core Eukaryotic Genes Mapping Approach (CEGMA) [49] identified 239 complete and 242 complete and partial core eukaryotic genes, estimating the assembly completeness at 96.4 % or 97.6 %. We predict 10,134 protein-coding genes resulting in 10,307 protein models, of which 9,476 have support from RNA, making the size and number of protein models similar to that of the beetle pathogen, *T. inflatum*, which is 30.3 Mb and has 9,998 protein models. They both also share high GC contents, 57.3 % and 58 %, for *T. ophioglossoides* and *T. inflatum*, respectively. These species are very closely related, which is reflected in the genome scale phylogeny (Fig. 1) and the large regions of shared synteny (Fig. 2). There are, however, two large scale inversions and some small rearrangements with disagreement, suggesting these species have diverged despite the relatively short branch lengths separating them in the species phylogeny (Fig. 1). The large number of small non-syntenic points in the mummerplot alignment could be due to the reliance on short read technology and the presence of short contigs in both assemblies.

**Table 1:** Genome statistics for *Tolypocladium ophioglossoides* compared to *T. inflatum* (Bushley et al. 2013)

	*T. ophioglossoides*	*T. inflatum*
Size (Mb)	31.2	30.3
# Scaffolds	172	101
N50	668,222	1,509,745
Longest Scaff.	2,309,933	3,562,345
%GC	57.3	58
Protein-coding genes	10,134	9,998
# SM clusters	38	38

### An overview of secondary metabolites in *T. ophioglossoides*

The *T. ophioglossoides* and *T. inflatum* genomes harbor 45 and 55 core secondary metabolite genes – NRPS, PKS, terpene synthase, and DMATs – spread across 38 and 38 secondary metabolite gene clusters, respectively. Similar to the contrast observed between closely related *Metarhizium* species ([46]), *T. ophioglossoides* and *T. inflatum* differ in the types of core secondary metabolite genes they possess with 21 shared between the two species, 34 unique to *T. inflatum*, and 24 unique to *T. ophioglossoides* (Additional file 1). Notably, *T. ophioglossoides* does not contain the NRPS gene, or any of the other genes in the *simA* cluster responsible for the production of cyclosporin A in *T. inflatum* [48]. However, *T. ophioglossoides* does share a Hypocreales-conserved core set of genes that flank the *simA* region with *T. inflatum*. Cyclosporin production has been reported from several species of *Tolypocladium* [50, 51], however, none of the truffle parasites, including *T. ophioglossoides*, have been demonstrated to produce the compound. Whether possession of the *simA* gene is a derived character state, or whether there has been a single or multiple losses within the genus, remains unknown and requires further sampling.

Secondary metabolites core genes predicted from the *T. ophioglossoides* genome include 15 PKSs, two PKS-like genes, 15 NRPSs, six NRPS-like genes, three hybrid NRPS-PKS genes, and four terpenes (Additional file 1). No DMAT synthases were identified in the *T. ophioglossoides* genome. Based on A-domain homology, two putative siderophore synthetases (one intracellular [TOPH_02853] and one extracellular [TOPH_02629]) were among the predicted NRPSs (Additional files 2 and 1). This is in contrast to *T. inflatum*, which possesses three putative siderophore synthetases (two extracellular and one intracellular) [48]. The entire Pseurotin-A precursor synthetase hybrid NRPS-PKS cluster (TOPH_07102) was identified in the *T. ophioglossoides* genome. Pseurotin-A, an antifungal compound described from several *Aspergillus* spp. [Eurotiales, Ascomycota], was also recently identified in the genome of *M. robertsii* [52]. The disjunct distribution of this secondary metabolite cluster raises several questions about the evolutionary mechanisms (*e.g*., horizontal gene transfer vs. complex patterns of gene loss) that may have led to this distribution.

*T. ophioglossoides* possesses the destruxins synthetase NRPS gene (TOPH_08872) (Additional file 1). Destruxins are known for their insecticidal properties in *Metarhizium* spp. [53], and the entire destruxins synthetase cluster was characterized by Wang et al. (2012) in *M. robertsii*. Homologs of the other essential genes in the destruxins cluster are present in the *T. ophioglossoides* TOPH_08872 cluster, except for *dtxS4* (Additional file 3), an aspartic decarboxylase responsible for producing β-alanine, one of the amino acids incorporated into destruxins. There are inversions in this cluster between *M. robertsii* and *T. ophioglossoides* as well, including the *dtxS2* aldo-keto reductase homolog (TOPH_08871) and an ABC transporter (TOPH_08869), which was not found to be essential in destruxins production in *M. robertsii* [54]. For these reasons, it is not likely that *T. ophioglossoides* produces destruxins, but possibly produces another group of related compounds. The sequenced strain of *Tr. virens* shares a homolog of the destruxins NRPS gene (Tv62540) (Additional file 3), but destruxins have not been reported to be produced by that species either [18].

To date, only two secondary metabolites have been reported to be produced by *T. ophioglossoides*: ophiocordin (also reported as balanol) and ophiosetin [55–57]. Ophiosetin is structurally similar to equisetin, an antibiotic with inhibitory activity of HIV-1 integrase, and both are produced by NRPS-PKS hybrid genes. Based on phylogenetic analysis of A-domains, cluster synteny with the equisetin cluster [58], and sequence homology, this study identifies the putative ophiosetin synthetase cluster around the hybrid NRPS-PKS, TOPH_07403 (Additional file 1). Further studies involving transformations and chemical verification and characterization of this cluster will be necessary to confirm this genotype-chemotype linkage. Ophiocordin is a polyketide and no putative gene or gene cluster related to its production was identified here. Except for the two peptaibiotic clusters discussed below, the remaining 33 secondary metabolite gene clusters are not yet associated with a specific gene product.

### Peptaibiotics of *Tolypocladium*

**Fig. 3:**
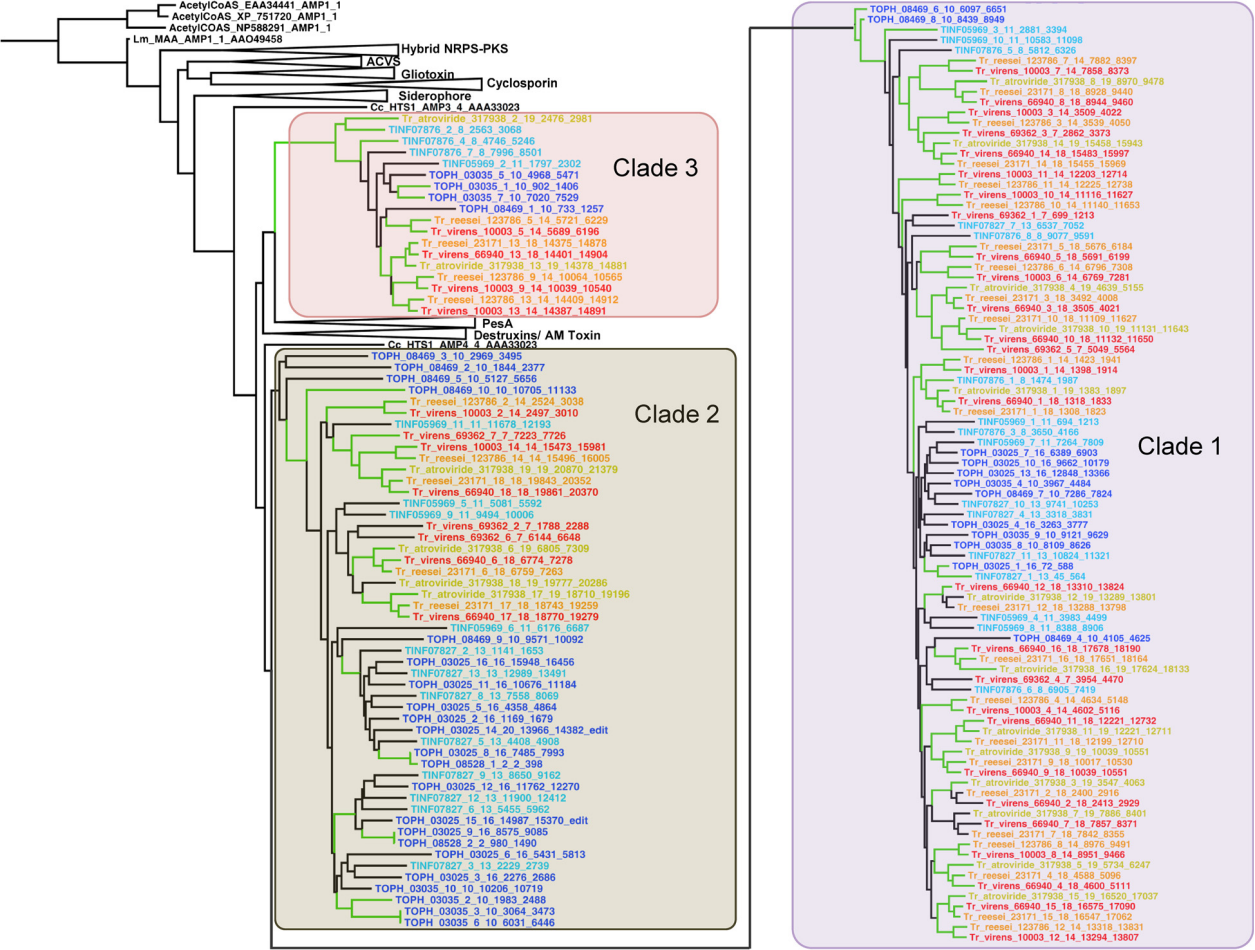
Phylogeny of the three peptaibol A-domain clades. Peptaibol focused, A-domain phylogeny created using RAxML, showing the 3 major clades. Green branches represent those supported by ≥70 % bootstrap support. *Tolypocladium* spp. A-domains are colored in blue and teal, and *Trichoderma* spp. A-domains are colored in yellow, red, or orange

Among the wide assortment of secondary metabolite genes and gene clusters, the draft genome of *T. ophioglossoides* possesses four peptaibiotic NRPS genes located within three gene clusters. Phylogenetic analyses of fungal A-domains from a variety of NRPS genes known to produce specific products and from whole genome mining of the hypocrealean species in Fig. 1, revealed that *Trichoderma* peptaibol A-domains group into three clades, one of which (Clade 3) is well supported in all analyses (Fig. 3, Additional file 2), the other two clades have strong support with the exception of a few A-domains from one of the *T. ophioglossoides* peptaibiotic genes (TOPH_08469) (see below). All A-domains from the four peptaibiotic genes in *T. ophioglossoides* fall within these three clades (Fig. 3), and representation in the three peptaibol clades is exclusive to A-domains from *Trichoderma* and *Tolypocladium. T. inflatum* also possesses three peptaibiotic NRPS genes. Strikingly, this limits the presence of peptaibiotic A-domains and genes to the two sampled mycoparasitic lineages in Hypocreales.

There are differences between species and gene membership within A-domain clades, however. For instance, Clade 1 is enriched in A-domains from the *Trichoderma* peptaibols (62 *Trichoderma* A-domains vs. 27 *Tolypocladium* A-domains), while Clade 2 contains more A-domains from *Tolypocladium* spp. (17 *Trichoderma* A-domains vs. 33 *Tolypocladium* A-domains). Importantly Clade 1 contains A-domains that encode for incorporation of AIB, as well as other A-domains that encode for incorporation of isovaline, leucine, isoleucine, alanine, glycine, valine, and serine ([21, 35]). Clade 2 is known to include A-domains that encode for valine, glutamine, asparagine, leucine, and isoleucine ([21, 35]). Clade 3, which is known only to incorporate a single amino acid, proline ([21, 35]), has a relatively equal distribution of both *Trichoderma* (10) and *Tolypocladium* (8) A-domains. Prolines are proposed to play an important structural role in peptaibols by creating a kink in the peptaibol chain, and Clade 3 occupies a long branch within the tree, suggesting it is highly diverged from the other A-domains (Additional file 2).

Peptaibols were reported from *T. geodes* [59] based on chemical isolation, but all previous reports of the compounds were identified from fungi in Hypocreaceae [29] (or Boletaceae [Basidiomycota] which are likely produced by hypocreaceous mycoparasites of Boletaceae fruiting bodies [60, 61]). A gene cluster responsible for the production of these peptaibols has not been identified, and to date no genomic sequence data have been produced for *T. geodes* from which to predict which genes or clusters may be responsible for its production. Efrapeptins, which are peptaibiotics originally described from *T. inflatum*, have been reported from several species of *Tolypocladium* [38], and it remains unknown whether the products produced by these clusters in *T. ophioglossoides* are of the efrapeptin class, or more traditional class of peptaibols. Regardless, the phylogenetic diversity of peptaibiotic NRPSs, as revealed by phylogenomic analyses of A-domains, supports a greater chemical diversity of peptaibiotics than currently known from chemical analyses.

**Fig. 4:**
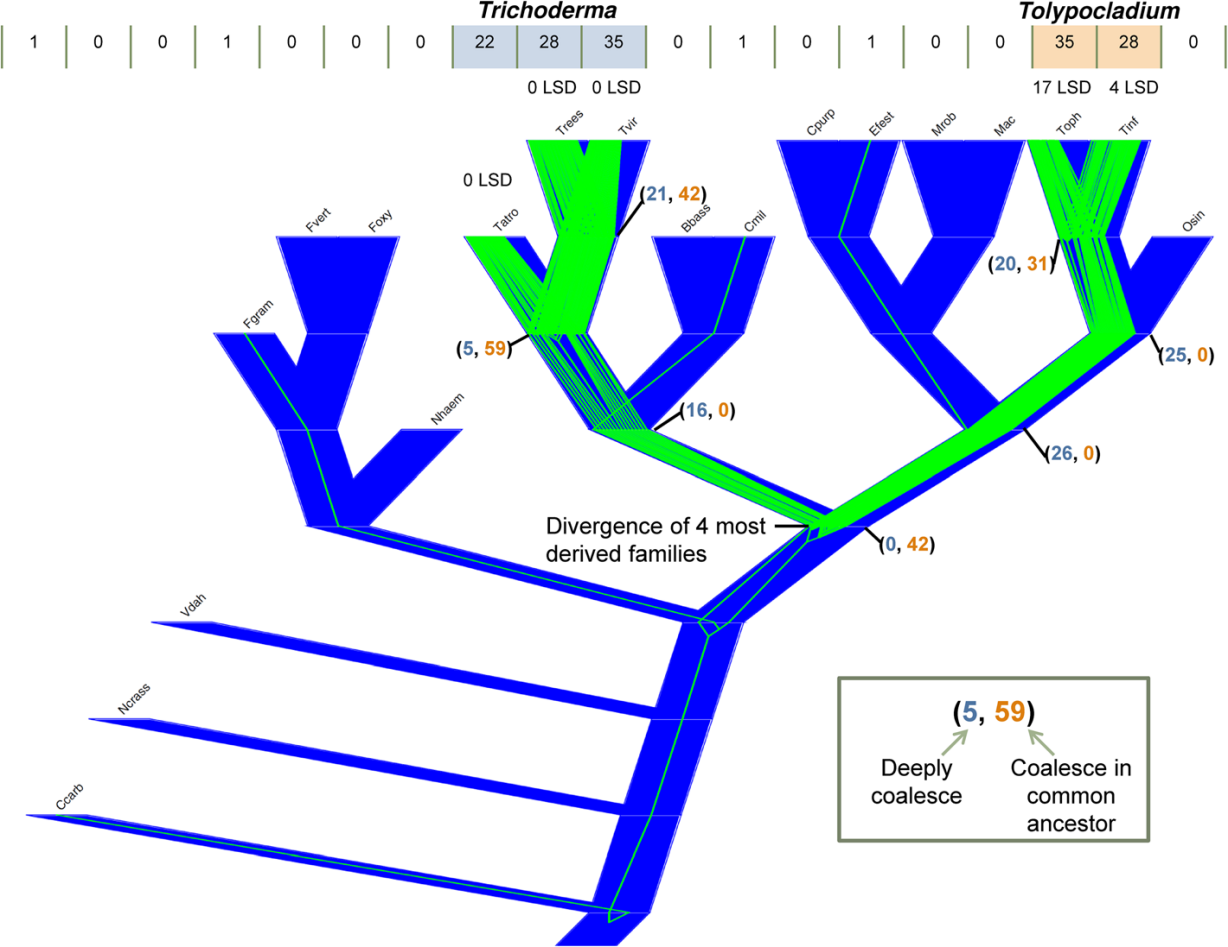
A-domain tree/species tree reconciliation. Reconciliation of the peptaibiotic A-domain (clades one and two) tree with the species tree. See Additional file 4 for clade 3 reconciliation. To root the tree, closely related outgroup A-domains from *Cochliobolus carbonum* (Ccarb), *Fusarium graminearum* (Fgram), *Cordyceps militaris* (Cmil), and *Epichloë festucae* (Efest) were included. Other abbreviations include: Neurospora crassa (Ncrass), *Verticillium dahliae* (Vdah), *F. verticillioides* (Fvert), *F. oxysporum* (Foxy), *Nectria haematococcum* (Nhaem), *Tr. atroviride* (Tatro), *T. reesei* (Trees), *Tr. virens* (Tvir), *Beauveria bassiana* (Bbass), *Claviceps purpurea* (Cpurp), *Metarhizium robertsii* (Mrob), *M. acridum* (Mac), *T. ophioglossoides* (Toph), *T. inflatum* (Tinf), *Ophiocordyceps sinensis* (Osin)

To interpret the evolutionary history of the peptaibol A-domains within Hypocreales, the peptaibol A-domain clade tree was reconciled with the species tree (Fig. 4). Because Clade 3 is inferred to have an independent origin from Clades 1 and 2 within the A-domain phylogeny, it was reconciled with the species tree separately (Additional file 4). We have included closely related A-domains from a complete A-domain phylogeny of Hypocreales that are not a part of the three peptaibol A-domain clades in order to root the peptaibol A-domain clades. The deep coalescence of peptaibol A-domains from all three clades at the common ancestor of *Trichoderma* and *Tolypocladium* (Fig. 4) suggests that the presence of peptaibiotics in *Tolypocladium* is an ancient attribute of *Tolypocladium* genomes and not a product of a more recent horizontal transfer. Analyzing Clades 1 and 2, only 31 of the A-domains in *T. ophioglossoides* and *T. inflatum* coalesce at their most recent common ancestor, whereas 20 coalesce more deeply at the divergence of the four most derived families in Hypocreales (Fig. 4).

There exist at least two possible explanations to these findings in *Tolypocladium* spp. First, the divergence of the A-domains could be significant enough so as to distort the evolutionary history as represented in the phylogenetic tree, especially since many of the branches of the *Tolypocladium* A-domain tree do not have strong bootstrap support. This explanation is not supported by the data, because based on the species phylogeny amino acid divergence levels between *Tolypocladium* species is less than that of *Trichoderma* species (Fig. 1). Second, incomplete lineage sorting could lead to this pattern of coalescence, suggesting that the ancestor to the genus possessed a multitude of these A-domains within one or several peptaibiotic genes that have undergone a complex history of ancient gains (duplications) and losses.

In contrast, five of the A-domains inferred to be present in the common ancestor of *Trichoderma* spp. deeply coalesce, and 59 are shared in the common ancestor. This pattern indicates a higher degree of domain tree – species tree congruence, which could be explained by two different mechanisms including 1) vertical descent with expansion and maintenance or 2) horizontal gene transfer from *Tolypocladium* to common ancestor of *Trichoderma*. To test for signatures of horizontal gene transfer, the A-domain tree was reconciled, with a modified species tree in which *Trichoderma* was sister to *Tolypocladium*, and outgroup A-domains were included to root the domain tree. This produced a smaller deep coalescent cost (212 v. 275), but this is due to the fewer number of extinctions (in Cordycipitaceae, Clavicipitaceae, and *O. sinensis*) required (118 v. 181), because the number of duplications remained the same (91 v. 91). In this simulated reconciliation, 14 A-domains were inferred to deeply coalesce, and of these, seven were inherited in each lineage. Taken together, this suggests that the diversity of *Trichoderma* A-domains cannot solely be characterized as the product of horizontal transfer from *Tolypocladium* A-domains. Instead it suggests that the common ancestor of *Trichoderma* possessed a small number of peptaibiotic NRPSs and A-domains that largely diversified in a manner consistent with speciation of the genus.

### *T. ophioglossoides* peptaibiotic gene clusters

**Fig. 5:**
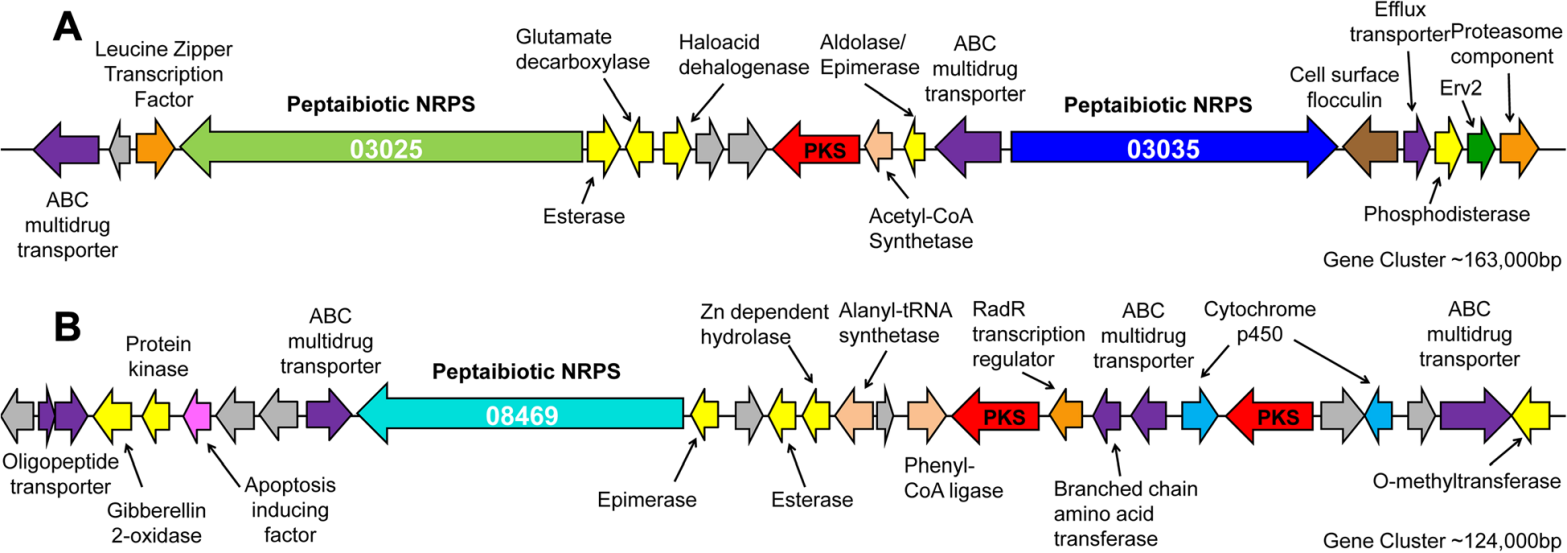
Large peptaibiotic gene clusters of *T. ophioglossoides*. Panel **a** shows T. ophioglossoides scaffold 5 that includes two peptaibiotic NRPS genes, while panel **b** shows the other large NRPS gene which lies scaffold 45. These clusters were predicted by the antiSMASH pipeline, and putative annotation of genes within clusters are given. Proteins without putative functions are colored gray, and sizes of genes and spacing are approximate in scale

**Fig. 6:**
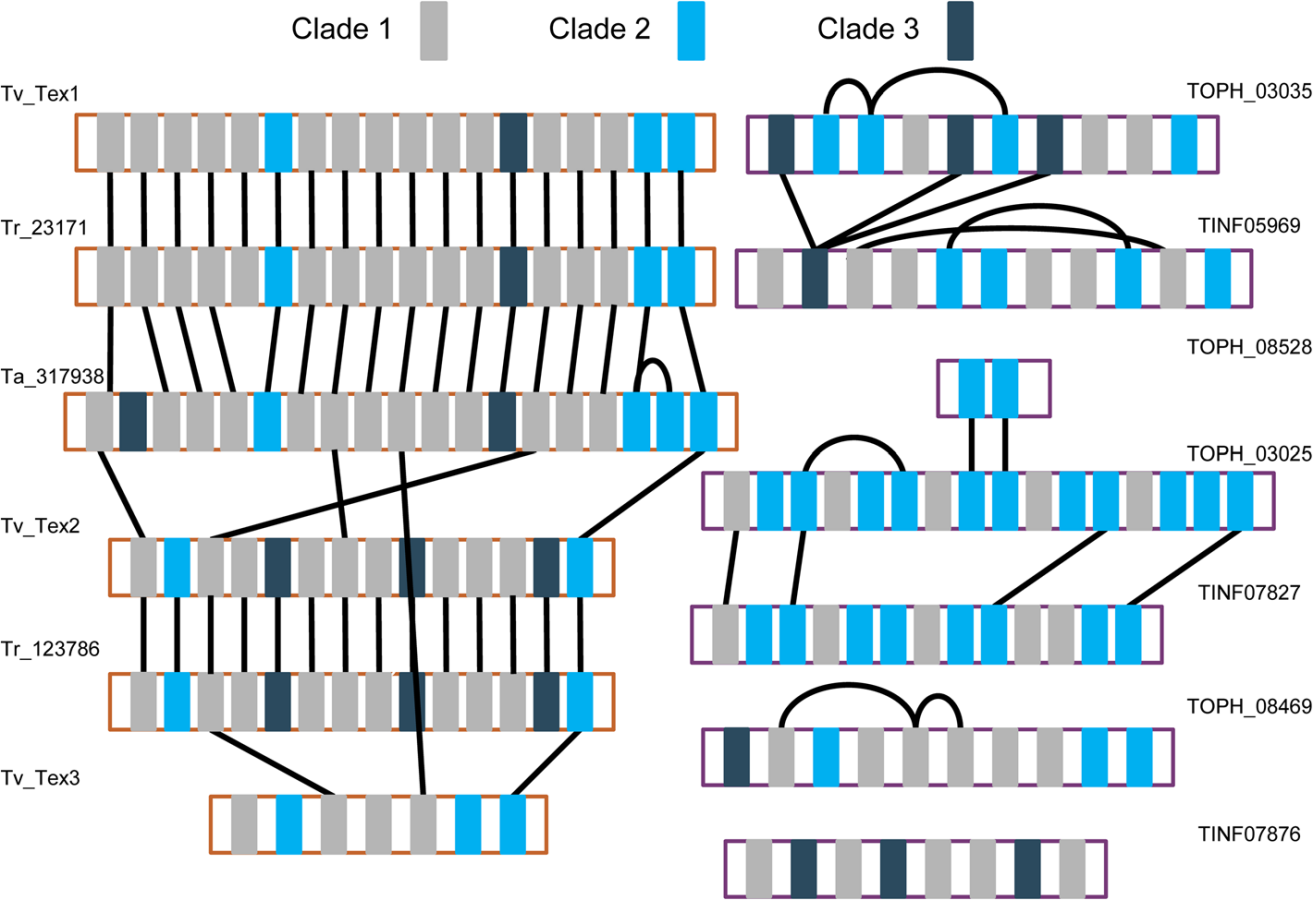
A-domain synteny map of for peptaibiotic genes in *Tolypocladium* and *Trichoderma*. Black lines connect orthologous A-domains as determined by MLBP ≥50 % in the reduced peptaibol A-domain tree (Fig. 2)

The four peptaibiotic genes (TOPH_03025, TOPH_03035, TOPH_08469 and TOPH_08528) are located in three gene clusters on three different scaffolds (Fig. 5, Additional file 1). Genes TOPH_03035 (10 modules) and TOPH_03025 (16 modules) are located within the same gene cluster and separated by only nine genes, including one PKS. In total, the cluster contains 18 genes, many of which are typically found within secondary metabolite clusters including two multidrug transporters (TOPH_03022 and TOPH_3034), a decarboxylase (TOPH_03027), an esterase (TOPH_03026), an epimerase (TOPH_03033), and a leucine zipper transcription factor (TOPH_03024) among others (Fig. 5a). At more than 17,000 amino acids in length, TOPH_03025 encodes for the largest peptaibiotic NRPS produced by *T. ophioglossoides*, and is the largest gene (52.5 kb) in the genome. Gene TOPH_08528 (2 modules), located on a separate scaffold, is only 2,587 amino acids in length and shares a high degree of amino acid identity with modules 8 and 9 of TOPH_03025 (Fig. 6). The genes surrounding TOPH_08528 include those typically found in an intact secondary metabolite cluster, including an ABC transporter, transcription factors, and an esterase, and there are no indications that TOPH_08528 is a nonfunctional pseudogene. However, no short peptaibiotics have been described so far, but the two A-domains of this gene both fall within peptaibiotic Clade 2 (Fig. 3), and it is clearly orthologous to part of TOPH_03025. So while it may not be producing a peptaibiotic, it is analyzed here only within the evolutionary context of these genes.

The remaining *T. ophioglossoides* peptaibiotic NRPS gene, TOPH_08469 (10 modules), is located within a cluster containing two PKS genes (TOPH_08457 and TOPH_08462), several ABC transporters (TOPH_08453, TOPH_08459, and TOPH_08470), an esterase (TOPH_08466), an epimerase (TOPH_08468), a hydrolase (TOPH_08465), two cytochrome p450s (TOPH_08458 and TOPH_08455), and a RadR transcription regulator (TOPH_08461) (Fig. 5b). It remains to be seen if the products of the PKSs are incorporated into the peptide created by TOPH_08469. Some of the A-domains within TOPH_08469 are divergent (Fig. 3), especially those that group (without support) as the earliest diverging lineages of Clade 2, and the presence of these A-domains in the tree, causes the support for this clade to weaken substantially.

### Peptaibiotic cluster synteny between mycoparasitic and insect pathogenic *Tolypocladium* spp.

**Fig. 7:**
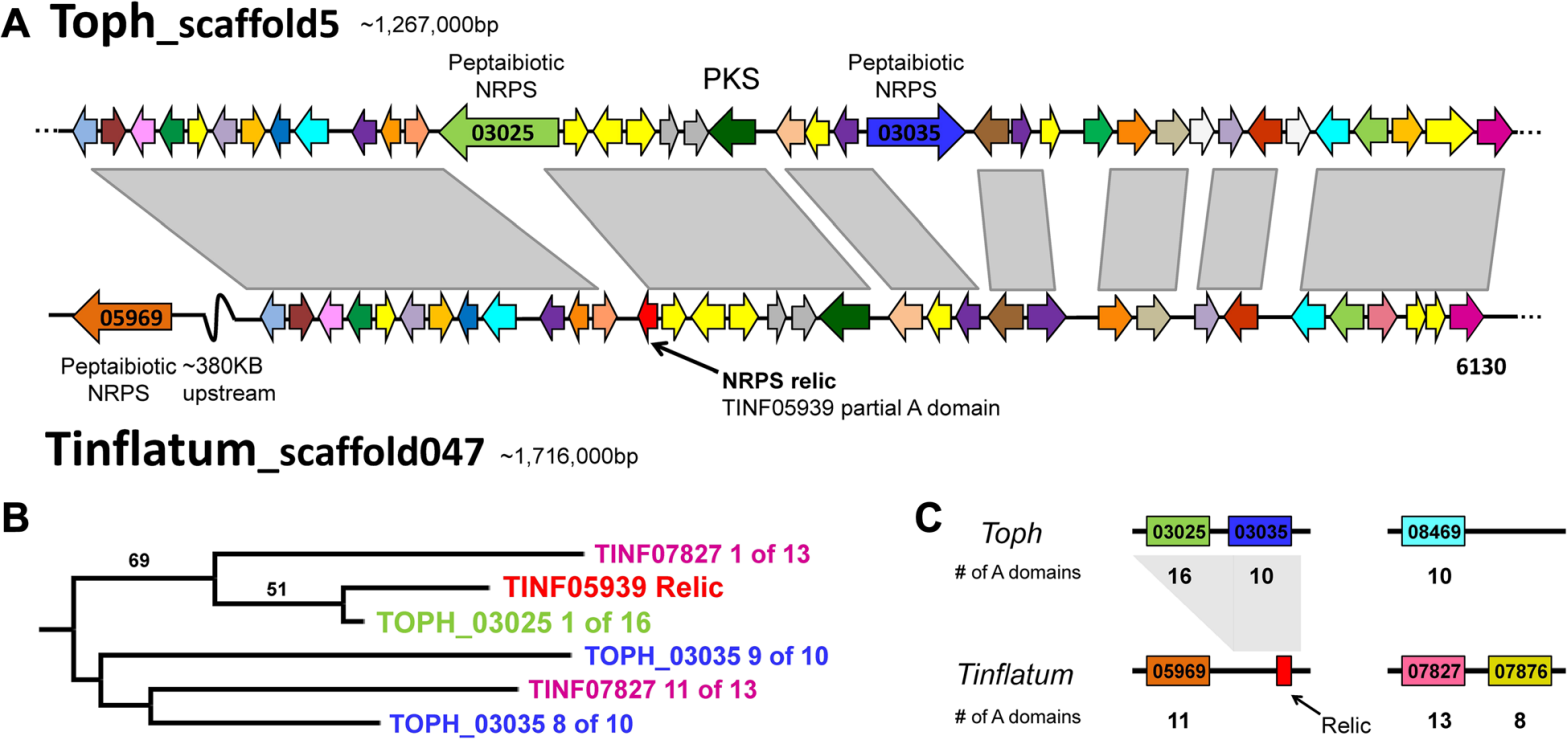
Analysis of cluster containing two peptaibiotic genes in *T. ophioglossoides* and corresponding region of the *T. inflatum* genome. **a**. Alignment showing high degree of synteny between *T. ophioglossoides* scaffold containing two peptaibiotic genes,TOPH_03025 and TOPH_03035, and scaffold 047 in *T. inflatum*, which also contains the peptaibiotic gene TINF05969, that is over 380 kb upstream of the syntenic region. **b**. Subsection of the large A-domain tree showing the relationship between the TINF05939 relic NRPS and the first A domain of TOPH_03025. **c**. Map of major peptaibiotic genes within the *Tolypocladium* genomes with the number of modules per gene shown below the scaffold. Gray area represents the syntenic regions between these clusters

Despite also possessing three large peptaibiotic genes in two clusters, *T. inflatum* peptaibiotic genes are highly divergent from *T. ophioglossoides* and the gene clusters are not located in the same regions of the genome (Fig. 7c). Alignment of *T. ophioglossoides* scaffold containing two peptaibiotic NRPS genes (TOPH_03025 and TOPH_03035) with the *T. inflatum* scaffold containing the single peptaibiotic NRPS gene (TINF05969), revealed high synteny in the *T. ophioglossoides* two-peptaibiotic gene cluster region, except for the absence of the two peptaibiotic genes, themselves (Fig. 7a). No other genes are present on the *T. inflatum* scaffold in the positions where the peptaibiotic NRPS genes are located in the *T. ophioglossoides* genome. This alignment did reveal, however, the presence of a truncated N terminal portion of an NRPS (TINF05939) that aligns with the N terminal region of TOPH_03025. When included in the entire hypocrealean A-domain phylogeny from Additional file 1, this *T. inflatum* NRPS “relic”, TINF05939, is most closely related to the first A-domain of TOPH_03025 (Fig. 7b). While retrotransposon relics are reported from secondary metabolite gene clusters [62, 63] and secondary metabolite gene modules may be truncated [25], this is the first report of a relic NRPS domain that remains within an otherwise intact secondary metabolite cluster including the PKS located within the cluster. Due to the truncated nature of this protein model (only 68 amino acids in length) and lack of other functional domains, it is unlikely that this relic produces a secondary metabolite peptide. There is no evidence of transposable elements in or near this cluster in either of the *Tolypocladium* genomes. As mentioned above, this *T. inflatum* scaffold which is syntenous with the gene cluster in *T. ophioglossoides* containing two peptaibiotic NRPS genes, also contains the NRPS gene TINF05969, but this gene is located approximately 380 Kb downstream of the relic cluster (Fig. 7a).

The region of the scaffold containing the *T. ophioglossoides* third large peptaibiotic gene, TOPH_08469, does not align well with any portion of the *T. inflatum* genome. Similarly, the final peptaibol cluster in *T. inflatum*, containing two peptaibiotic NRPS genes (TINF07827 and TINF07876), does not align well to any portion of the *T. ophioglossoides* genome. The lack of synteny between these clusters in *Tolypocladium* spp. highlights the significant amount of genomic rearrangements between these closely related taxa. Campbell et al. [64] observed patterns of differential gene loss in *Botrytis* spp. within an ancient, horizontally-transferred, secondary metabolite gene cluster, leading to a patchy distribution of the genes within the clusters. This is not the pattern seen in the peptaibiotic clusters in *Tolypocladium* spp., in which the protein models are not reciprocal best BLAST hits (except for the protein models in the “relic” cluster in *T. inflatum*). Thus, despite the fact that their products may have similar functions, these peptaibiotic NRPS genes are divergent and located within nonhomologous gene clusters.

### Mixed homology of peptaibol A-domains in Hypocreales

Using the moderate to strongly supported nodes (≥50 maximum likelihood bootstrap percentage [MLBP]) in the A-domain phylogeny as a guide for module homology, the peptaibol NRPS genes are more conserved among the *Trichoderma* species examined as compared to species of *Tolypocladium* (Fig. 6), a finding reflected in the domain tree – species tree reconciliation analyses (Fig. 4). Using whole genome data of the sampled species of *Trichoderma*, A-domains from the *Tr. virens* three peptaibol NRPS genes (*tex1*, *tex2*, and *tex3*) [65] were identified for the phylogenetic analyses; *Tr. virens* peptaibols contain 18, 14, and 7 modules respectively. *Tr. reesei* has two peptaibol NRPSs (Tr_23171 and Tr_123786) which possess 18 and 14 modules. In the annotation of *Tr. atroviride* IMI 206040, the A-domain HMM identified one 19 module peptaibol NRPS (Ta_317938), and several single A-domain protein models that group within the three peptabioitic clades and are all located on scaffold 29. Further examination of this *Tr. atroviride* gene region using the JGI genome browser (Grigoriev et al. 2014) revealed that all of the *ab initio* gene predictions of that region predict a single protein model that is the approximate length of the 14 module peptaibol genes in *Tr. virens* and *Tr. reesei*. Degenkolb et al. [36] identified a homolog of the 14 modular peptaibol gene from a different strain of *Tr. atroviride*, and thus this is likely a mis-annotation of *Tr. atroviride* scaffold 29. Alignment of this scaffold in *Tr. atroviride* (scaffold 29) to those of *Tr. virens* and *Tr. reesei* (Additional file 5) revealed high nucleotide homology. However, the flanking regions did not align well, and a BLAST search of the *Tr. atroviride* genome using the nucleotide sequences from *Tr. virens* and *Tr. reesei* revealed that the 14 modular peptaibol gene in *Tr. atroviride* is located within a different portion of the genome than in the other two *Trichoderma* spp. (Additional file 5).

Comparing the *Trichoderma* A-domains, each species possesses one ortholog of the large 18 or 19 module NRPS gene (Fig. 6), and at the nucleotide level these regions of their genomes also align and are syntenous (Additional file 5). The A-domains are syntenous in their arrangement within the large peptaibol NRPS genes across the species, except for: (a) the insertion of a Clade 3 domain at the third module, and (b) a duplication of either the seventeenth or eighteenth A-domains, which are most closely related. Within the two 14 module NRPS genes in *Tr. virens* and *Tr. reesei*, there is complete synteny of the A-domains. In *Tr. virens*, it has been demonstrated that this 14 module NRPS, Tex2, is responsible for two different sizes of peptaibols (11 and 14 residues in length) [35]. Due to differences in annotation (see above), the 14 module peptaibol gene in *Tr. atroviride* is not compared in this analysis. The short 7 module peptaibol synthetase from *Trichoderma* spp. is found only in *Tr. virens*. Between these three groups of peptaibol NRPSs, the terminating residues are all orthologous, as well as the initiating residues in the larger classes of peptaibol NRPSs.

In *Tolypocladium*, there is a very different pattern of homology and synteny between the peptaibiotic NRPSs of the two species. Only a few of the A-domain relationships within *Tolypocladium* are statistically supported (≥50 MLBP) (Figs. 3 and 6). The first, third, and last A-domains of the largest NRPS in both species (TOPH_03025 and TINF07827) are orthologous, but not the other domains within those two genes. There are several instances of intragenic module duplications which are known to occur within NRPS genes and have been proposed to play a role in the evolution of novel metabolites [23]. Within TOPH_03035, for example, there is strong support for a shared ancestry between modules 2, 3 and 6 (Fig. 6), indicating that these modules are the product of lineage specific duplications (Fig. 4). This indicates a more complicated evolutionary history of these genes in *Tolypocladium*.

The lack of module synteny and orthology between *Tolypocladium* peptaibiotic gene modules is comparable to the lack of genomic synteny observed between their clusters. Part of this is due to the deep coalescence of the *Tolypocladium* A-domains. This evidence indicates that *Tolypocladium* peptaibiotic genes are not highly similar but are the products of more ancient divergences. This is notable, because in contrast to *Trichoderma* spp., all of which exhibit some degree of mycoparasitism (Druzhinina et al. 2011), *T. ophioglossoides* and *T. inflatum* have different ecologies, which are characterized by mycoparasitism and insect pathogenicity, respectively. Thus, if peptaibols are important in successful mycoparasitism (as the case has been made in *Trichoderma* spp. [30, 31], then there may be less selective pressure to maintain a specific mycoparasitic function of these extremely large (>10,000 amino acid) NRPS genes in more ecologically diverse lineages. Future genome-scale studies sampling other hypocrealean lineages containing both mycoparasites and insect pathogens, including the genus *Polycephalomyces*, could further test this hypothesis.

## Conclusions

The genome of *T. ophioglossoides* is rich in secondary metabolite gene clusters, and 31 out of 38 of these clusters have no putative product. Given this potential and its life history as a mycoparasite, this species should be targeted for future studies to discover novel natural compounds with potential antibiosis, including antifungal, activity. The *simA* NRPS gene cluster, responsible for the production of the immunosuppressant cyclosporin, is not present in the *T. ophioglossoides* genome, but three large peptaibiotic genes are present within two clusters. These are the first data to suggest the potential for peptaibiotic production from a mycoparasitic species of *Tolypocladium*. This study confirms the presence of three phylogenetic clades of peptaibiotic NRPS A-domains from *Tolypocladium* and *Trichoderma* spp., and that peptaibiotics in general are limited to the mycoparasitic lineages of Hypocreales, based on current sampling. Reconciliation of the A-domain tree with the organismal phylogeny reveals that the peptaibiotic NRPSs of *Trichoderma* and *Tolypocladium* are likely the product of different mechanisms of diversification. *Trichoderma* is characterized by A-domain diversification that is largely consistent with speciation whereas *Tolypocladium* is characterized by A-domain diversity that shows patterns of deep coalescence. Deep coalescence is inconsistent with peptaibiotic NRPS diversity being the product of HGT to *Tolypocladium*, rather it is the product of complex patterns of lineage sorting and gains and losses of A-domains from hypocrealean ancestors. While the diversity of peptaibiotic NRPSs in *Trichoderma* could possibly be explained by HGT in the common ancestor of the three species, none of the *Tolypocladium* peptaibiotic NRPSs analyzed here are candidates for HGT. Further research is required to identify the structures of specific metabolites of the *Tolypocladium* gene clusters and to determine if these peptaibiotics are produced during mycoparasitism by *T. ophioglossoides* or if these genes are present in other mycoparasitic lineages of Hypocreales.

## Methods

### Genome sequencing

*T. ophioglossoides* strain CBS 100239 was grown for 7 days in a shaking incubator in potato dextrose broth (PDB) inoculated with plugs of tissue growing on potato dextrose agar for collection of tissue for DNA extraction. Tissue was harvested via filtration, frozen at −80 °C in 1.5 mL tubes, and then lyophilized for 24 h. Lyophilized tissue was ground using a mortar and pestle, and DNA was extracted using a Qiagen DNeasy Plant Mini kit following the standard protocol starting at the step with the addition of lysis buffer AP1 and eluted in 50 μL water. Tissue for RNA extraction was grown in Yeast Malt (YM) broth, minimal media (MM) containing autoclaved insect cuticle with proteins removed using the protocol in Andersen (1980) [48], and MM containing lyophilized *Elaphomyces muricatus* peridium for 24 h and harvested into liquid nitrogen and stored at −80° until extraction. RNA was extracted using the Qiagen RNeasy Plant kit following the manufacturer’s protocol. The small insert DNA library was prepared using New England Biomedicals NEBNext reagents, and size selection (350 bp) was performed using gel extraction. Nextera Mate Pair Sample Preparation of a large insert (6800 bp) library and sequencing was conducted by the Core Labs at the Center for Genome Research and Biocomputing (CGRB) at Oregon State University. The Illumina TruSeq RNA Sample Preparation Kit v2 was using for RNA library construction, using the manufacturer’s suggested protocols including Agencourt AMPure magnetic beads for cleaning steps. All libraries were sequenced on the Illumina HiSeq2000 at the Core Labs of the CGRB with paired-end 101 cycles for DNA libraries and single-end 51 cycles for RNA libraries.

### Assembly, annotation, and bioinformatic analyses

Using scripts in the fastx toolkit [66], raw reads were trimmed (to 50 bp in length) and filtered based on quality score (all bases ≥ q20). Initial de novo assembly of the short insert reads was conducted in Velvet v. 1.19 [67] with over 156 million reads where the assembly had a median coverage depth of 74.45. The final trim length (50 bp) used in the assembly was chosen after trimming to different lengths (40–80 bp) followed by quality filtering and then the assembly with highest n50 and fewest number of contigs was selected as the “best” assembly. From that assembly, 50 million overlapping 150 bp paired reads were simulated with a 250 bp insert size using the program wgsim v. 0.3.1-r13 in “haploid” mode [68]. Final assembly using the simulated overlapping short insert library reads and the mate pair reads from the 6 kb library was conducted in AllPaths-LG with default settings [69]. The Core Eukaryotic Mapping Genes Approach (CEGMA) was used to estimate the completeness of the *T. ophioglossoides* genome [49]. Scripts in the Mummer3 package were used to create a mummerplot between *T. ophioglossoides* and *T. inflatum*; specifically nucmer v. 3.07 was run with default settings [70].

Gene model predictions were created using the Maker annotation pipeline [71] incorporating RNA data assembled in Trinity [72] using the Jellyfish v. 2.0 method of kmer counting [73]. Other information given to Maker included a custom hidden Markov model (HMM) for *T. ophioglossoides* built by Genemark-ES v 2.0 [74], a SNAP HMM [75] trained on *Fusarium graminearum*, which was also set as the species model for AUGUSTUS [76], and protein and/or EST data from the following hypocrealean taxa: *F. graminearum*, *N. haematococca*, *Tr. reesei*, *Tr. virens*, *M. robertsii*, *T. inflatum*, *C. militaris*, *B. bassiana*. Annotation of transposable elements was performed in RepeatMasker v 3.2.8 with organism set to “fungi” [77], and custom repeat content was estimated using RepeatScout v 1.0.3 and scripts associated with that package [78]. Non-overlapping *ab initio* protein models were aligned using BLAST [79] against a custom database of all the protein models of all the hypocrealean taxa used in this study. Any of these protein models with a significant hit (≤ 1e^−5^) were included in the final protein set and used for downstream analyses.

Using a set of NRPS A-domains from a wide array of published fungal genomes [20] an HMM was created for this study using the program Hmmer 3.0 [80]. This HMM was then used to mine the 18 hypocrealean genomes used for this study for the identification of A-domains. Putative A-domains identified were filtered for short sequences (less than 100 bp), and where applicable cross referenced with published reports of NRPS from those species (*e.g*., *Tr. virens* Tex1) [34]. Additional annotation of secondary metabolite clusters was completed using the antiSMASH [81] and SMURF [82] pipelines. A-domain trees were reconciled with species trees in Mesquite v. 2.75 with the contained tree treated as unrooted [83].

Whole scaffold alignments were performed in the program Mauve [84] with default progressivemauve alignment settings.

### Phylogenetic analyses

Predicted A-domain amino acids sequences were aligned using MUSCLE v 3.8.31 [85] under default settings. Gaps were removed manually, and all alignments were analyzed using RAxML v 7.2.6 [86] using the Gamma model of rate heterogeneity and the WAG substitution matrix with 100 bootstrap replicates.

Whole genome phylogenomic analyses were executed in the HAL pipeline [87]. Orthologous clusters of proteins were identified in MCL [88] across inflation parameters 1.2, 3 and 5. Briefly, orthologous clusters were filtered for retention of clusters with one sequence per genome and removal of any redundant clusters. The resulting unique, single-copy orthologous clusters of proteins were aligned in MUSCLE [85] with default settings; poorly aligned regions were identified using Gblocks ([89]; gap removal setting = c, for conservative) and excluded from subsequent analyses. The aligned clusters were concatenated into a superalignment and maximum likelihood analysis was performed using RAxML v 7.2.6 with the Gamma model of rate heterogeneity and the WAG substitution matrix with 100 bootstrap replicates.

### Availability of supporting data

This Whole Genome Shotgun project has been deposited at DDBJ/EMBL/GenBank under the accession LFRF00000000. The version described in this paper is version LFRF01000000.

## Additional files

Additional file 1:
**Table of secondary metabolite genes and clusters in** ***T. ophioglossoides*** **.** Genes belonging to the same clusters are highlighted in the same color. Cluster prediction based on antiSMASH. Siderophores are marked as either intracellular (i) or extracellular (e). Orthologs of *T. inflatum* (*Ti*) were annotated as reciprocal best BLAST hits and further based on amino acid alignment and A-domain phylogeny. (DOCX 22 kb) Additional file 2:
**RAxML phylogeny of A-domains mined from the hypocrealean genomes samples (Fig.** 1 **).** A-domains from *T. ophioglossoides* are highlighted in blue. Taxonomic abbreviations: *Alternaria alternata* (Aalt), *Aspergillus fumigatus* (Afu), *A. nidulans* (Anid), *Beauveria bassiana* (Bbass), *Claviceps purpurea* (Cpurp), *Cochliobolus carbonum* (Ccarb), *Cordyceps militaris* (CCM), *Epichloë festucae* (Efest), *Fusarium equiseti* (Fe), *F. graminearum* (FGSG), *F. heterosporum* (Fh), *F. oxysporum* (FOXG), *F. verticillioides* (FVEG), *Gibberella fujikuroi* (Gf), *Leptosphaeria maculans* (Lmac), *Magnaporthe grisea* (MGG), *Metarhizium acridum* (MAC), *M. robertsii* (MAA), *Nectria haematococca* (Nhaem), *Neurospora crassa* (Ncrass), *Ophiocordyceps sinensis* (Osin), *Penicillium chrysogenum* (PC), *Rhizopus oryzae* (RO), *Tolypocladium inflatum* (TINF), *T. ophioglossoides* (TOPH), *Trichoderma atroviride* (Tr_atro), *Tr. reesei* (Tr_reesei), *Tr. virens* (Tr_virens), *Ustilago Maydis* (UM), *Verticillium dahliae* (VDAG). (PDF 57 kb) Additional file 3:
**Destruxins NRPS and cluster in** ***T. ophioglossoides*** **and other taxa.** A. Excerpts from larger A-domain phylogeny (Additional file 2) showing the phylogenetic relationships of destruxins A-domains (which group into two areas within the A-domain phylogeny; the PerA clade, and then *T. inflatum simA* clade) in *M. robertsii*, *T. ophioglossoides*, and *Tr. virens*. B. Nucleotide alignment of *M. robertsii* destruxins cluster contig with homologous region in *T. ophioglossoides* genome. Abbreviations as in Additional file 2. (TIFF 409 kb) Additional file 4:
**A-domain Clade 3 tree/species tree reconciliation.** Reconciliation of the peptaibiotic A-domain tree with the species tree. Abbreviations as in Fig. 4. (TIFF 1364 kb) Additional file 5:
**Alignment of** ***Trichoderma*** **peptaibol scaffolds.** Top panel depicts alignments of the regions of Trichoderma genomes that contain the large (18–19 module) peptaibol genes (which are boxed in blue). Bottom panel shows alignments of scaffold containing the *Trichoderma* 14 module peptaibol genes. one demonstrates that the region around the 14 module peptaibols in *Tr. reesei* and *Tr. virens* are conserved and syntenic with *Tr. atroviride* contig 25, which does not contain an NRPS. Alignment two shows the same two scaffolds in *Tr. reesei* and *Tr. virens*, but aligned with *Tr. atroviride* contig 29, which does not share synteny with the other two species except for the peptaibol NRPS. (TIFF 10788 kb)
