# Supplementary material for: The genome of the truffle-parasite Tolypocladium ophioglossoides and the evolution of antifungal peptaibiotics
Source: BMC Genomics. 2015 Jul 28;16(1):553. doi: 10.1186/s12864-015-1777-9 (PMC4517408; doi:10.1186/s12864-015-1777-9)
Supplement: Additional file 1: — Table of secondary metabolite genes and clusters in T. ophioglossoides . Genes belonging to the same clusters are highlighted in the same color. Cluster prediction based on antiSMASH. Siderophores are marked as either intracellular (i) or extracellular (e). Orthologs of T. inflatum (Ti) were annotated as reciprocal best BLAST hits and further based on amino acid alignment and A-domain phylogeny. (DOCX 22 kb) [file 12864_2015_1777_MOESM1_ESM.docx]

**Additional file 5 – Table of secondary metabolite genes and clusters in *T. ophioglossoides*.** Genes belonging to the same clusters are highlighted in the same color. Cluster prediction based on antiSMASH. Siderophores are marked as either intracellular (i) or extracellular (e). Orthologs of *T. inflatum* (*Ti*) were annotated as reciprocal best BLAST hits and further based on amino acid alignment and A domain phylogeny.

| Gene | Description | anti-SMASH | SMURF | hmm A domain? | # A domains predicted | Putative product | *Ti* ortholog? |
| --- | --- | --- | --- | --- | --- | --- | --- |
| TOPH_00033 | NRPS-like | x |  | yes | 1 |  |  |
| TOPH_00044 | PKS | x | x |  |  |  | x |
| TOPH_00667 | PKS-like |  | x |  |  |  | x |
| TOPH_01006 | PKS | x | x |  |  |  | x |
| TOPH_01395 | PKS | x | x |  |  |  | x |
| TOPH_01399 | PKS | x | x |  |  |  | x |
| TOPH_01953 | Terpene | x |  |  |  |  |  |
| TOPH_02629 | NRPS | x | x | yes | 1 | Siderophore (e) | x |
| TOPH_02853 | NRPS | x | x | yes | 3 | Siderophore (i) | x |
| TOPH_02894 | PKS | x | x |  |  |  | x |
| TOPH_03025 | NRPS | x | x | yes | 16 | Peptaibiotic |  |
| TOPH_03031 | PKS | x | x |  |  |  | x |
| TOPH_03035 | NRPS | x | x | yes | 10 | Peptaibiotic |  |
| TOPH_03217 | NRPS-like |  | x |  |  |  |  |
| TOPH_03419 | NRPS | x | x | yes | 4 |  | x |
| TOPH_03459 | NRPS-like | x | x |  |  |  | x |
| TOPH_03628 | Terpene | x |  |  |  |  |  |
| TOPH_04325 | Terpene | x |  |  |  |  |  |
| TOPH_04431 | PKS | x | x |  |  |  |  |
| TOPH_05458 | PKS | x | x |  |  |  | x |
| TOPH_05817 | NRPS | x | x | yes | 3 |  | x |
| TOPH_06344 | NRPS-Like | x | x |  |  |  | x |
| TOPH_06345 | PKS |  | x |  |  |  | x |
| TOPH_07065 | PKS | x | x |  |  |  |  |
| TOPH_07087 | PKS | x | x |  |  |  | x |
| TOPH_07102 | Hybrid | x | x | yes | 1 | Pseurotin-A |  |
| TOPH_07403 | Hybrid | x | x | yes | 1 | Ophiosetin |  |
| TOPH_07700 | PKS | x | x |  |  |  |  |
| TOPH_07813 | NRPS | x | x | yes | 2 |  |  |
| TOPH_08016 | PKS | x | x |  |  |  |  |
| TOPH_08020 | PKS | x | x |  |  |  |  |
| TOPH_08068 | Terpene | x |  |  |  |  |  |
| TOPH_08168 | PKS-like |  | x |  |  |  |  |
| TOPH_08247 | Hybrid | x | x | yes | 1 |  | x |
| TOPH_08386 | NRPS-like | x | x | yes | 1 |  | x |
| TOPH_08411 | NRPS | x |  | yes | 1 |  |  |
| TOPH_08457 | PKS | x | x |  |  |  | x |
| TOPH_08462 | PKS | x | x |  |  |  |  |
| TOPH_08469 | NRPS | x | x | yes | 10 | Peptaibiotic |  |
| TOPH_08528 | NRPS | x | x | yes | 2 | Homology to Peptaibiotic genes |  |
| TOPH_08794 | NRPS | x | x |  |  |  | x |
| TOPH_08872 | NRPS | x | x | yes | 6 | Destruxin |  |
| TOPH_08946 | NRPS-like | x | x |  |  |  | x |
| TOPH_09389 | NRPS | x | x | yes | 2 |  |  |
| TOPH_09714 | NRPS | x | x | yes | 2 |  |  |
