# Supplementary figures and images for: The genome of the truffle-parasite Tolypocladium ophioglossoides and the evolution of antifungal peptaibiotics
Source: BMC Genomics. 2015 Jul 28;16(1):553. doi: 10.1186/s12864-015-1777-9 (PMC4517408; doi:10.1186/s12864-015-1777-9)

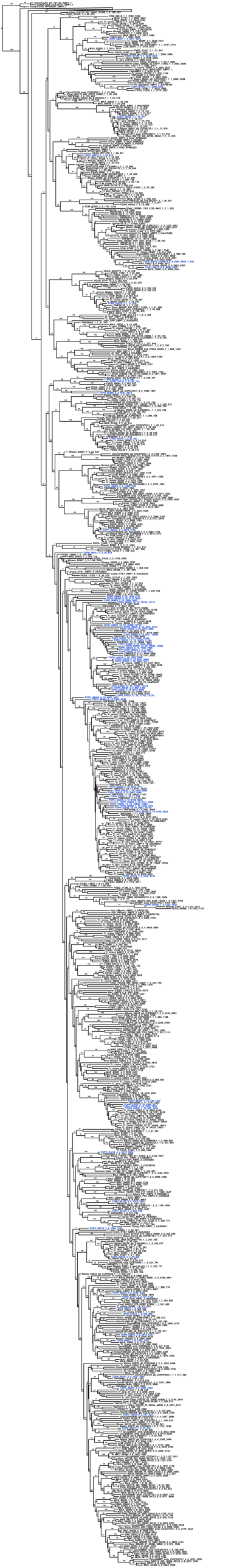

Supplement: Additional file 2: — RAxML phylogeny of A-domains mined from the hypocrealean genomes samples (Fig. 1 ). A-domains from T. ophioglossoides are highlighted in blue. Taxonomic abbreviations: Alternaria alternata (Aalt), Aspergillus fumigatus (Afu), A. nidulans (Anid), Beauveria bassiana (Bbass), Claviceps purpurea (Cpurp), Cochliobolus carbonum (Ccarb), Cordyceps militaris (CCM), Epichloë festucae (Efest), Fusarium equiseti (Fe), F. graminearum (FGSG), F. heterosporum (Fh), F. oxysporum (FOXG), F. verticillioides (FVEG), Gibberella fujikuroi (Gf), Leptosphaeria maculans (Lmac), Magnaporthe grisea (MGG), Metarhizium acridum (MAC), M. robertsii (MAA), Nectria haematococca (Nhaem), Neurospora crassa (Ncrass), Ophiocordyceps sinensis (Osin), Penicillium chrysogenum (PC), Rhizopus oryzae (RO), Tolypocladium inflatum (TINF), T. ophioglossoides (TOPH), Trichoderma atroviride (Tr_atro), Tr. reesei (Tr_reesei), Tr. virens (Tr_virens), Ustilago Maydis (UM), Verticillium dahliae (VDAG). (PDF 57 kb) [file 12864_2015_1777_MOESM2_ESM.pdf]

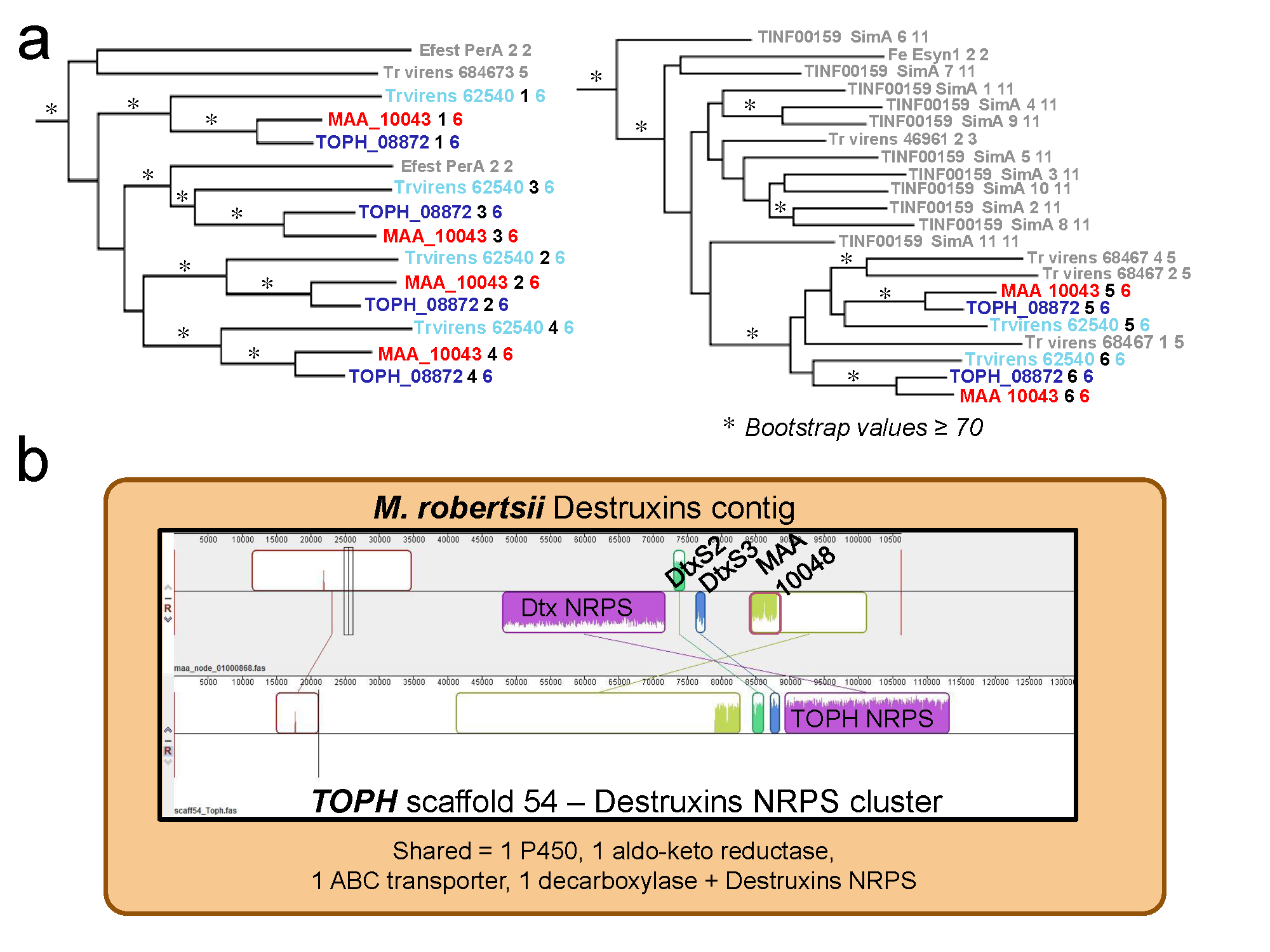

Supplement: Additional file 3: — Destruxins NRPS and cluster in T. ophioglossoides and other taxa. A. Excerpts from larger A-domain phylogeny (Additional file 2) showing the phylogenetic relationships of destruxins A-domains (which group into two areas within the A-domain phylogeny; the PerA clade, and then T. inflatum simA clade) in M. robertsii, T. ophioglossoides, and Tr. virens. B. Nucleotide alignment of M. robertsii destruxins cluster contig with homologous region in T. ophioglossoides genome. Abbreviations as in Additional file 2. (TIFF 409 kb) [file 12864_2015_1777_MOESM3_ESM.tiff]

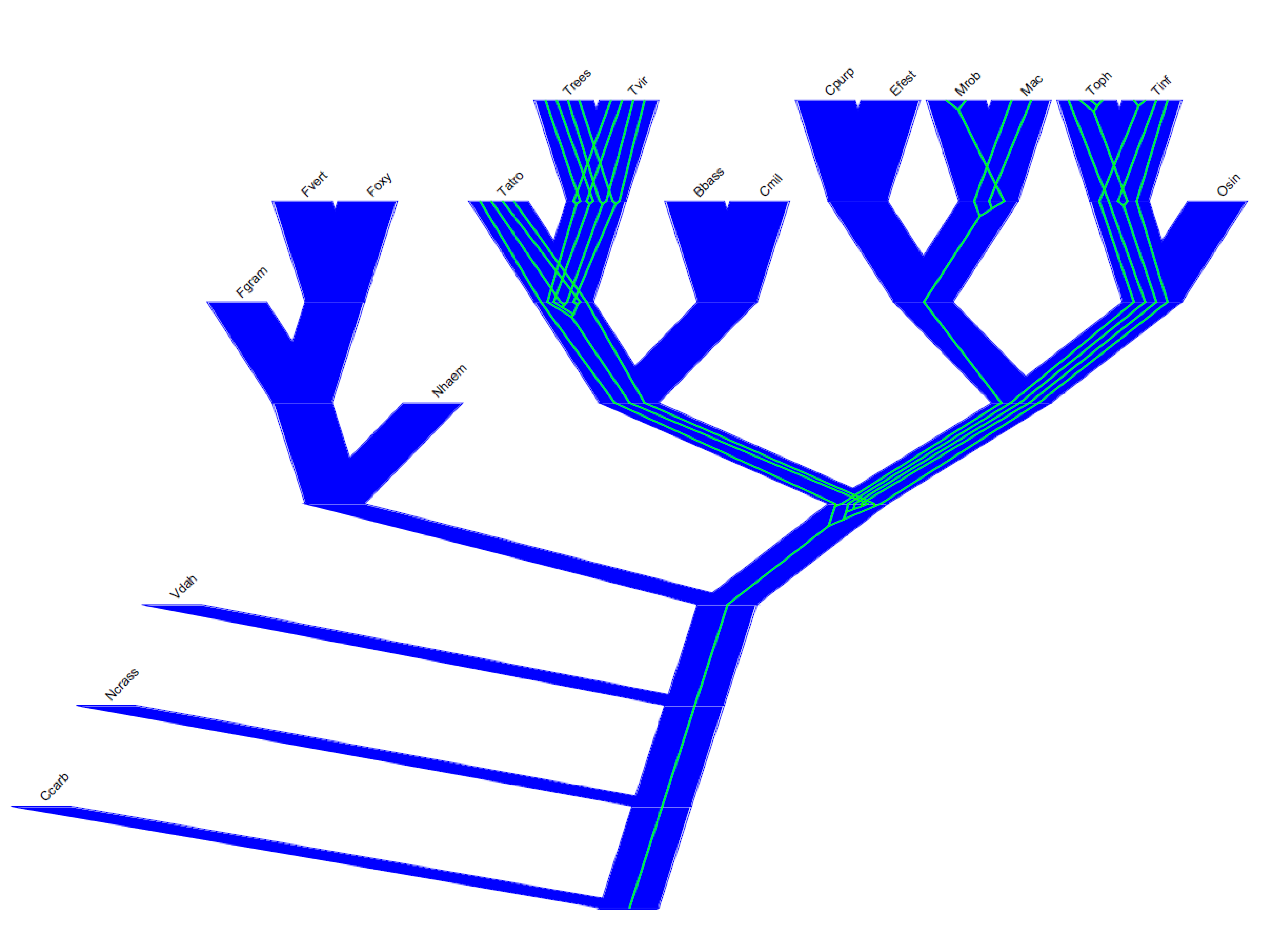

Supplement: Additional file 4: — A-domain Clade 3 tree/species tree reconciliation. Reconciliation of the peptaibiotic A-domain tree with the species tree. Abbreviations as in Fig. 4. (TIFF 1364 kb) [file 12864_2015_1777_MOESM4_ESM.tiff]

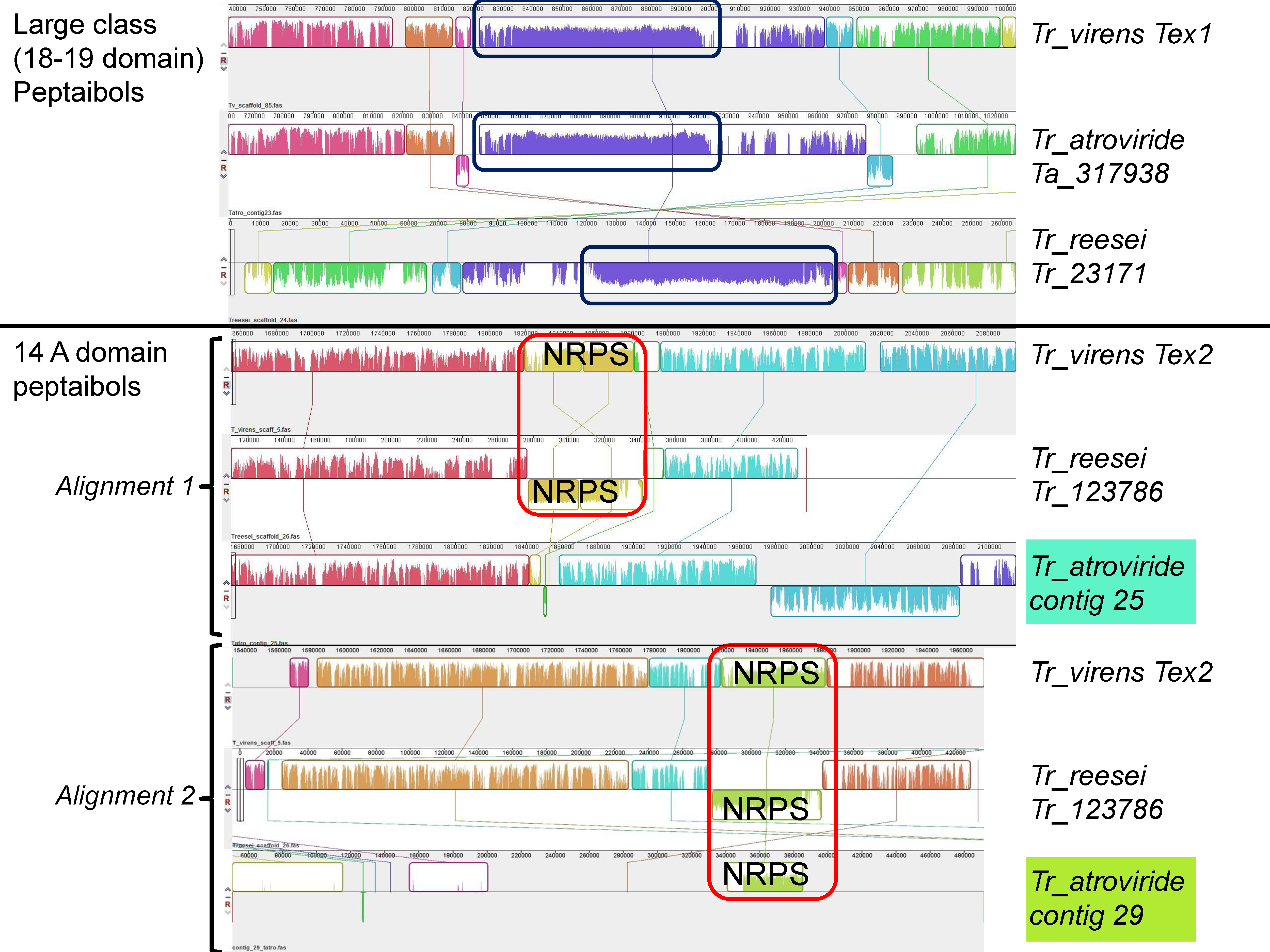

Supplement: Additional file 5: — Alignment of Trichoderma peptaibol scaffolds. Top panel depicts alignments of the regions of Trichoderma genomes that contain the large (18–19 module) peptaibol genes (which are boxed in blue). Bottom panel shows alignments of scaffold containing the Trichoderma 14 module peptaibol genes. one demonstrates that the region around the 14 module peptaibols in Tr. reesei and Tr. virens are conserved and syntenic with Tr. atroviride contig 25, which does not contain an NRPS. Alignment two shows the same two scaffolds in Tr. reesei and Tr. virens, but aligned with Tr. atroviride contig 29, which does not share synteny with the other two species except for the peptaibol NRPS. (TIFF 10788 kb) [file 12864_2015_1777_MOESM5_ESM.tiff]
